# Nutrition Interventions for Prevention and Management of Childhood Obesity: What Do Parents Want from an eHealth Program?

**DOI:** 10.3390/nu7125546

**Published:** 2015-12-15

**Authors:** Tracy Burrows, Melinda Hutchesson, Li Kheng Chai, Megan Rollo, Geoff Skinner, Clare Collins

**Affiliations:** 1 School of Health Sciences, Faculty of Health, University of Newcastle, Newcastle 2308, Australia; Melinda.hutchesson@newcastle.edu.au (M.H.); likheng.chai@uon.edu.au (L.K.C.); megan.rollo@newcastle.edu.au (M.R.); Clare.Collins@newcastle.edu.au (C.C.); 2 Priority Research Centre for Physical Activity and Nutrition University of Newcastle, Newcastle 2308, Australia; 3 School of Design, Communication and IT, University of Newcastle, Newcastle 2308, Australia; Geoff.skinner@newcastle.edu.au

**Keywords:** healthy lifestyle program, families, online, Internet, eHealth, childhood obesity

## Abstract

With the growth of Internet technologies, offering interventions for child and family weight management in an online format may address barriers to accessing services. This study aimed to investigate (i) whether an eHealth family healthy lifestyle program would be of interest to parents; and (ii) preferences and/or expectations for program components and features. Parents of children aged four to18 years were recruited through social media and completed an online survey (54 items) including closed and open-ended questions. Responses were collated using descriptive statistics and thematic analysis. Seventy-five participants were included (92% mothers, mean age 39.1 ± 8.6 years, mean BMI 27.6 ± 6.3 kg/m^2^). The index child had a mean age of 11 ± 6.2 years with 24% overweight/obese. The majority of parents (90.3%) reported interest in an online program, with preference expressed for a non-structured program to allow flexibility users to log-on and off as desired. Parents wanted a program that was easy to use, practical, engaging, endorsed by a reputable source, and able to provide individual tailoring and for their children to be directly involved. The current study supports the need for online delivery of a healthy lifestyle program that targets greater parental concerns of diet rather than child weight.

## 1. Introduction

Published studies which have examined interventions aimed at prevention of childhood obesity [1], showed successful strategies to include parents as the agent of change [2] and provision of resources to parents to encourage lifestyle changes within the home [1]. Whilst current prevention and treatment measures are effective in the short-term (<6 months), there is insufficient evidence to assess long-term effectiveness [1,3]. Future interventions need to be adaptable to family routines that acknowledge the increased busy-ness with more parents now in the workforce and be adaptable to various settings in order to be adopted and sustainable in the longer-term [1].

Family units and the home environment are recognised to have the greatest influence on child lifestyle habits and behaviours and hence serve as targets for prevention and treatment of obesity during childhood [4]. Parents have been acknowledged as the primary influence on the development of child eating and physical activity behaviours with their parenting styles also playing a role in development of healthy lifestyles [5]. Parents are therefore recognised as the key agents of change [4].

A large proportion of treatment interventions to date have been developed through a researcher or health practitioner perspective and have not considered or consulted with families to identify their needs and views despite being well recognised in health promotion planning and evaluation cycles [6,7]. Suggestions have been made to move from efficacious interventions towards, involving parents directly in the development phase of the program to ensure better effectiveness and translation [7].

With the growing use of and access to the Internet [8] in addition to a plethora of mobile applications (apps) targeting child health [9], delivery of web-based/online interventions and use of apps have been identified as a direction that should be tested in the delivery of family focused healthy lifestyle programs [10,11]. The use of web-based intervention may address the issues related to working parents and busy lifestyles, which can be a barrier to participating in healthy lifestyle programs. Ownership of smartphones is increasing with penetration rates continueing to increase in Australia [12], suggesting that many parents would own a smartphone. Over 31% of Australian children 5–14 years own a smartphone, with this figure increasing to 76% for 12–14 years olds [13]. Evidence suggests that 57% of US parents who use apps reported downloading apps for their children to use [14]. However, what is currently available in the online arena largely serves only for those already overweight/obese and some not based on efficacious strategies. For example in a recent review of dietary apps available for use with children showed that the majority of these apps were not evidence based [9].

Therefore, the aim of this exploratory study was to investigate whether parents of children aged four to 18 years would be interested in a healthy lifestyle program that would be directed at the family unit and delivered using technology. A secondary aim was to determine what components parents thought should be included in such a program and the desired format.

## 2. Experimental Section

### 2.1. Participants and Setting

Data for the current cross-sectional survey were collected online to inform design of a healthy lifestyle intervention with a focus on diet for families delivered using technology. Parents of children aged 4 to 18 years residing in Australia and fluent in English were recruited using convenience sampling. An email which contained a link to the survey was sent by researchers to university staff and students mailing lists and promoted through a variety of social networking platforms including the University of Newcastle’s (UoN) Facebook page, the UoN blog, and Hunter Medical Research Institute webpage. This study used “virtual snowballing” with participants asked to share the survey link with others in the target group via email and/or social networking sites to increase size and access to families who are an increasingly hard group to reach with more parents now in the workforce. All participants provided informed consent after reading the information statement and eligibility questions were answered. Upon completion of survey, participants had the option of entering a prize draw to win one of five shopping vouchers, valued at 100 AUD each. The study was approved by the University of Newcastle’s Human Research Ethics Committee.

### 2.2. Data Collection

A 54-item online survey was adapted from a previous survey [15] and tailored to the current study by asking more specific questions regarding diet and family oriented questions. The survey was developed and released using the Survey Monkey online platform with responses sent to researchers via a secure, encrypted connection. Respondents contact details were stored separately from survey data, making the responses anonymous. The survey was pilot tested to check for readability, flow, spelling, grammar and ambiguity in questions with only minor changes required to achieve improved flow and readability.

The survey asked parents demographic questions about themselves and one *index child* only, chosen by the parent and within the specified age range of 4 to 18 years. The survey took approximately 15 min to complete and comprised of nine open-ended questions and 45 fixed responses with the majority of questions having an “other” category where participants could provide additional free text responses if they wished. Participant responses from the survey were grouped into the five survey categories as follows:

### 2.3. Demographic Characteristics (12 Items)

Participant demographic data included highest qualification, marital status, ethnicity, number of children in the household and postcode, which was used to determine the Index of relative Socioeconomic Advantage and Disadvantage (ISRAD) where postcode is rated from one to 10, with one being an area of most disadvantage/least advantage and 10 being least disadvantaged/most advantaged [16]. Participants were asked if they had regular access to the Internet, defined as daily/weekly and the location of access (*i.e.*, home, work, mobile phone). Data were collected about parents and the index child’s date of birth, gender, height and weight, which was converted to BMI using standard equations and then for parents classified as underweight healthy, or overweight based on BMI less than 18.5 kg/m^2^, between 18.5 and 24.9 kg/m^2^, or more than 24.9 kg/m^2^, respectively. For children anthropometric values were used to generate BMI *z* scores using least-mean-square (LMS) methods [17] and then classified using international cut points for each sex [18].

### 2.4. Weight Status and Perceptions (19 Items)

Parents were asked if they felt their child’s weight needed to be changed with options of yes, no or unsure. If they answered “yes” parents were then asked about possible reasons for wanting to change their children’s weight, with response options including “to improve self-confidence”, “to change their appearance”, “to improve health”, “to reduce /avoid bullying” or “other”. Parents were asked if they had previously attempted strategies to change their child’s lifestyle (*i.e.*, diet and physical activity) in the previous 12 months and the specific resources and types of changes attempted. For resources, participants could select from six responses consisting of dietary books/manuals, web based programs, smart phone app, visiting a health professional, or selecting “other” and add a free text response. For types of changes attempted, participants could select from five options related to fussy eating, fruit and vegetable intake, snacking, and other, which allowed a free text response.

### 2.5. Participants’ Interest and Preferences for Content in a Proposed Program (23 Items)

Participants were asked to rate their level of interest in participating in a family healthy lifestyle program. Reponses were rated on a five point Likert scale ranging from “very interested” to “not at all interested”. Several questions were included in the survey to assess preferred program features and content and included methods shown to be effective in eHealth interventions [19]. Items included: methods for recording of family goals and lifestyle components, including weight and diet (e.g., email, mobile app, online); delivery of information (email, by a dietitian, able to be accessed at their discretion); nature of program being formalised (meet at a scheduled time online for a group session with other parents for a specific number of weeks) or informal (able to be access when and which components based on personal choice); involvement of children and how to engage them; child’s computer accessibility and proficiency. Participants could also indicate which topics would be of interest (options included goal setting, recipes, assessment tools, healthy eating) and were able to provide any other suggestions through open-ended responses. Participants were also asked open-ended questions to detail the aspects that would encourage them and their family members to participate in an online healthy lifestyle program or alternatively why they would not be interested in participating, or to indicate any potential barriers to participation.

### 2.6. Statistical Analysis

Descriptive statistics were used to analyse close-ended questions from the survey, chi squared analysis was used to determine differences by group (age and Socioeconomic disadvantage based on ISRAD). JMP 10.0 (SAS Institute) was used to calculate means for continuous variables and percentages for categorical variables. Data were reported as mean ± standard deviation and range where possible. Thematic analysis was used to examine open questions and responses to “other” options within closed questions. Responses were coded using an inductive method and the emergent themes identified and reported.

## 3. Results

A total of 115 people accessed the survey, eight participants did not meet the eligibility criteria with 113 providing consent, a total of 75 completed all survey questions and were included in the final analysis. Based on parent demographics variable (age, gender, and BMI), completers were not statistically different to non-completers (*p* > 0.05). The samples were predominantly mothers (*n* = 69) with a mean age of 39.1 ± 8.6 (range 23–54) years and the majority were classified as overweight with mean BMI of 27.6 ± 6.3 (range 19.5–51.1) kg/m^2^ (Table 1). The majority were married (72%); reported having one child or two children (80%), with 20% having three or more; and 38% had completed a certificate/diploma, while 42% had completed a university degree. Participants resided in areas of varying deciles based on the ISRAD scale, with 41% (*n* = 33) were ≤5 and 58% (*n* = 44) ≥6. Four per cent of participants identified as Aboriginal and/or Torres Strait Islander origin. The index child selected by parents was on average 10.7 ± 6.2 (4–18) years with approximately equal numbers of boys and girls (52% boys, *n* = 39). A total of 59% of children were classified as younger children (4–11 years) while 40% were classified as adolescent (12–18 years). Based on BMI *z* scores, a total of 37% were underweight, 39% were at healthy weight with 12% overweight and 12% obese. All participants reported having regular access to the Internet. The majority reported having their primary Internet connection at home (*n* = 60), followed by a mobile phone (*n* = 42) with all that had mobile access also having home access. At least one third of the participants reported having regular Internet access via multiple (>3) sources.

**Table 1:** Demographics of participants (*n* = 75) and their children.

	Range or *n*	Mean ± SD or %
**Age**	23–54	39.1 ± 8.6
**Parental role**		
Mother	69	92%
Father	6	8%
**Number of Children aged 4–18 years**		
1	29	39%
2	31	41%
3	10	13%
≥4	5	7%
**Highest Qualification**		
Higher University Degree (e.g., Grad Dip, Masters, PhD)	17	23%
University Degree	24	32%
Certificate/Diploma (e.g., childcare, technician)	21	28%
Trade/Apprenticeship (e.g., Hairdresser, Chef)	2	3%
Higher school certificate (Years 12 or equivalent)	8	11%
School certificate (Years 10 or equivalent)	3	4%
**Parent BMI**		
Underweight (BMI < 18.5 kg/m^2^)	0	0%
Normal (BMI 18.5–24.9 kg/m^2^)	32	43%
Overweight (BMI ≥ 25 kg/m^2^)	43	57%
Demographics of children		
**Age (years)**		
4–8	30	40%
9–11	14	19%
12–13	12	16%
14–18	19	25%
**Gender**		
Boy	39	52%
Girl	36	48%
**BMI *z* score**		
Underweight	28	37
Healthy weight	29	39
Overweight	9	12
Obese	9	12

### 3.1. Weight Status and Perceptions

For questions on participants’ concern and perception of their child’s weight, at least 79% reported not being concerned about their child’s current weight and <20% (*n* = 13) reporting they wanted to change their child’s weight. Parents were able to select more than one option for wanting to change child weight, the top three selected reasons were “to improve health” (*n* = 20), followed by “to reduce/avoid bullying” (*n* = 12) and “to improve self-confidence” (*n* = 10).

More than half of participants (55%) reported attempting to change their child’s dietary intake over the past 12 months. A total of 29 participants provided open-ended responses about which aspects they had attempted to change. The major themes included: adopting a general healthy diet (*n* = 11), followed by increasing vegetable intakes (*n* = 6), reducing sugar intakes (*n* = 2) and increasing fruit (*n* = 2). Most parents (63%) had not attempted to change their child’s physical activity over the same time frame, with the majority reporting their children as already being active.

### 3.2. Interest and Preferences for Proposed Program

Almost 90% of participants expressed interest in participating in an online healthy lifestyle program. The majority reported being “very interested” (*n* = 36) followed by “interested” (*n* = 18) and “somewhat interested” (*n* = 13). The majority of participants expressed preference for an informal program with no scheduled sessions (71%) as opposed to a formal online platform with structured modules (18%). The main preference for access to program content was online that included personal user accounts to access information. Participants preferences did not differ substantially (*p* > 0.05) by age (*i.e*., younger 4–11 years or adolescent 12–18 years), weight status of child or socioeconomic status (Table 2 and Table 3). Participants had a preference to enter family goals in lifestyle diaries via a website (52%) or smartphone app (57%). In contrast, reminders were preferred to be sent by email (45%) or SMS (67%) (Table 2). Close to half (45%) of the survey respondents also reported a desire to receive information about dietary intake from a qualified dietitian either via a face-to-face session (*n* = 16) or online (*n* = 18) (e.g., video call or blog site). About two-thirds of participants would prefer to interact with other program members and/or staff. The top two options were via social networking platforms and via an online forum embedded within the program website.

**Table 2:** Identified preferences of program content and contact by age group.

Preference for Record Keeping	Website %	Email %	Smartphone Application %	SMS %	Other %
**Family Goals**					
4–11 years	29	20	33	6	3 (iPad)
12–18 years	23	19	24	8	
Total	52	39	57	14	3 (iPad)
**Diary (website, smartphone app, notebook)**					
4–11 years	25	-	35	-	5 (Notebook/iPad)
12–18 years	25	-	24	-	9 (Notebook/iPad)
Total	50	-	59	-	14 (Notebook/iPad)
**Family weight record (sms, email, website, smartphone app)**					
4–11 years	19	9	23	12	3 (iPad)
12–18 years	21	15	25	9	1 refusal to enter child weight online
Total	40	24	48	21	4 (3)
**Monthly progress feedback (email, sms website, smartphone app)**					
4–11 years	20	32	21	9	
12–18 years	19	24	21	5	
Total	39	56	42	14	0
**Weekly motivational messages from staff (email, online blog, status update of social network group, sms)**					
4–11 years		32		20	7 (online blog), 20 (status update of social network group)
12–18 years		24		17	4 (online blog), 15 (status update of social network group)
Total	-	56	-	37	9 (online blog), 26 (status update of social network group)
**Reminders (email, sms, phone calls)**					
4–12 years		24		36	3
12–18 years		21		31	3
Total	-	45	-	67	5 (phone calls)

Expressed as % of responses of total sample, No differences between age groups *p* > 0.05.

**Table 3:** Identified preferences of program content and contact by socioeconomic status (ISRAD scale 0–10).

Preference for Record Keeping	Website %	Email %	Smartphone Application %	SMS %
**Family Goals**				
ISRAD 1–3	12	15	13	1
4–6	24	16	29	11
7–10	16	8	15	4
**Diary (website, smartphone app, notebook)**		Notebook		
ISRAD 1–3	13	3	15	
4–6	20	5	29	
7–10	17	4	15	
**Family weight record (sms, email, website, smartphone app)**				
ISRAD 1–3	12	8	16	8
4–6	16	8	23	11
7–10	12	9	9	3
**Monthly progress feedback (email, sms website, smartphone app)**				
ISRAD 1–3	9	15	16	4
4–6	15	28	17	7
7–10	15	13	9	4
**Weekly motivational messages (email, online blog, status update of social network group, sms)**				
ISRAD 1–3	-	15	5	8
4–6	-	25	4	17
7–10	-	16	4	12
**Reminders (email, sms, phone calls)**				
ISRAD 1–3		15		20
4–6		19		29
7–10		12		17

ISRAD—Index of relative Socioeconomic Advantage and Disadvantage, expressed as % of responses of total sample, no differences between groups *p* > 0.05.

For responses regarding program content, the most popular topics selected included: “Knowledge about healthy food portion sizes for different ages” (*n* = 56), “Healthy recipes” (*n* = 55), with “Specific information on nutrition topics” (*n* = 50) and “Education for my child about healthy eating” (*n* = 50) both ranked as the third most popular choices. Most participants wanted their child to be able to participate in the program in addition to the parents, with 52% (*n* = 39) reporting that the program should be inclusive of their child. Parental suggestions of how children could be included the use of online healthy eating games and activities, in place of accessing the information as parent(s). Most parents (*n* = 56) reported their child have regular access to mobile phones, and tablets. The themes identified from open ended questions by parents for inclusion were to: educate their child and to allow the child to hear the information from health professionals/other adults rather than parents being perceived as nagging. This is illustrated through the following quotes “To educate them so that they can take these tools into their future” and “kids …take personal responsibility along with the parent/family”. The participation was also identified as a means of support for the child. “I think it’s always easier to achieve goals as part of a group or a program, rather than going it alone”.

A total of 55 participants provided a comment in response to “What would encourage your family to participate?” The most common themes arising from participant responses were: (1) having a program that was easy to use and low cost; “It needs to be reasonably simple and streamlined, my family is quite busy and I imagine many other families are also”; (2) goal/goal setting specifically and for these to be time framed, achievable, being able to visually see and monitor progress “specific, measurable, achievable, reasonable with a timeframe on goals, rewarded in some way”; (3) be able to make content relevant for a wide range of family members; “ I think it needs to be able to cater for different child age groups I have kids ages 15, 9, 2. What is appropriate for the 15 years old will be too advanced for the 9 years old” and “The opportunity to have the program tailored to accommodate the whole family… meal planning and lifestyle changes are difficult in our house with so many different people’s like and dislikes”; (4) development of a website in addition to an app to increase accessibility and usage; “Making the app and a website to increase appeal to teenagers”; (5) development of a program that was not solely focused on weight but included feedback on diet “Feedback or advice on the current nutritional deficits in their regular diet. Weight is not a current concern for many families”; and (6) cost and time of health lifestyles with budget friendly meals/snacks ideas that used every day foods and “anything that gives tips and ideas to encourage particularly healthy eating and the tracking of this would be useful. Being time poor and not having great cooking ideas is our main issue” and “Make it easy to follow and not too many complicated foods”. Parents also reported that would be likely to engage in a program that was endorsed by a university or a recognised government body. “If I had an overweight child, if site was endorsed by union or government health department if low cost or free”.

Only a smaller group of parents (*n* = 8) reported not being interested in the program, mainly because they felt that their families/children were already healthy. This was elaborated through comments that included: “My children are active enough and of a healthy weight range” and “We are generally healthy in our lifestyle and prefer to be self-motivated”; “Time poor. It takes time to prepare healthy, nutritious food. Money - fresh produce is costly”, “Duration is my common barrier” and “a program that didn’t take up a great deal of daily time to complete the task involved”.

## 4. Discussion

The current study aimed to identify whether parents would be interested in participating in an online healthy lifestyle program designed for families and to determine the desired components, structures and features. The majority of participants expressed interest in participating in an online healthy lifestyle program for families and preferred it to be informal and in the form of a user friendly website in addition to a smart phone app.

Survey responses supported the development of an online healthy lifestyle program with a variety of content, including personalised information on healthy eating and portion sizes, which could be adapted and made relevant for different age groups to help engage all family members. In addition information on healthy recipes and recipe modification should be included. A previous focus group discussion on childhood obesity prevention found similar but broader themes of interest expressed by parents [20], including healthy lifestyle information and awareness, discrepancies between knowledge and behaviour, barriers to a healthy lifestyle, and prevention strategies for childhood obesity [20]. Current research suggests that parents need ideas and support on how to discuss healthy eating and physical activity in affirmative and motivating ways with their children [21]. Existing evidence supports the need for interventions centred on behaviour change rather than conveying facts using didactic approaches [22].

It has been highlighted previously that providing realistic targets for behaviour change and motivating parents to address child body weight were the key goals for future education programs [22]. In the current survey, the majority of parents were not concerned (less than 20%) about their children’s weight, however a larger proportion of parents expressed concern about their children’s dietary habits. These findings may relate to the weight status of the index children where approximately 37% of children were self-reported to be underweight and 39% healthy weight. These results differ from other studies where parents express no concern even when children are found to be overweight and obese [23]. Current systematic reviews report parents, especially those who were overweight themselves, have a tendency to misperceive their child’s weight [24]. It is crucial that parents recognise the importance of early intervention to reduce the risk factors for childhood obesity and its associated complications [24]. The increased concern for diet rather than weight found in this study provides rationale that one of the best targets for improving weight and lifestyle in children is via evaluating dietary intakes/patterns and targeting barriers to healthy eating and meal preparation. While self-reported height and weight via the web has been shown to be valid for adults [25], future research should consider how accurate parents are at self-reporting their children’s anthropometrics.

Further a substantial proportion of parents reported that their children were already active and did not need to change levels of physical activity, additional research is needed to ascertain if this perception is correct as current research demonstrates that a large proportion of children are not active enough [26]. Interventions that led participants to pertinent and personalised resources reported longer website session times per visit and more visits [27].

A family web-based intervention program (*n* = 18) targeting overweight children demonstrated that those with higher access and login rates were more likely to reduce their BMI *z* scores by approx. 0.05 unit and healthy eating behaviours over the four-week intervention period compared to those with low website usage rates [28]. This survey suggests that these higher access rates may be achieved through development of a website and smartphone app.

Many parents responding to the current survey specifically expressed a desire for their children to be involved in the program in order to expose them to nutrition education and healthy lifestyle information from an external authoritative source. Future intervention programs should be consider the age of the children targeted as there may be supervisory issues to consider with children in online environments. Parents indicated that they were concerned about food and food-related child behaviours [29]. In the current survey parents suggested an interactive website section, that incorporated child games and activities containing nutrition messages. Evidence also suggests that children need direct messages to motivate them to change diet and physical activity behaviours, as well as tips on cooperating with their parents to achieve lifestyle goals. For parent messages need to include how to talk about eating and exercise habits with their children in positive and encouraging ways, and to learn how to help their children maintain efforts to improve lifestyle behaviours [21]. Programs that have utilised both parents and children as agents of change have been shown to be effective in improving nutrition and weight outcomes, in both the short and long term [30,31,32]. Both parents and children report needing constructive and practical approaches to improve health, such as: achievable goals to healthy eating, healthy meals and recipes, ideas for physical games and activities the family can enjoy together, and referral services for local support groups [21]. It is vital for parents to participate with their children to prevent obesity and that families are supported through the use of effective tools to facilitate this cooperative effort [21]. Future studies should consider effective engagement and retention strategies for children of different age groups that occur within families, which was expressed by parents in this study. The core reasons for parents wanting a program that supported child weight management strategies were improved health, bullying prevention, followed by increased self-confidence.

In order to develop an effective online program to support families for improved lifestyle, potential barriers have to be identified in this study. Parents considered challenges to behaviour change, included time constraints and community pressures, as well as approaches to facilitating healthy food choices, including reward, education, and being creative with food [29]. The majority of parents in the current study wanted a flexible program with access to program content being convenient, without having to fit their schedule around formal program sessions. Similarly, parents of pre-school aged children from a study on the feasibility of developing an online program for obesity prevention reported that being able to access information in their own time was a priority [33], although they also wanted the program to be in the form of structured modules [33]. Parents in the current study preferred an informal program that allowed room for flexibility when assessing information. Parents also preferred to interact with other program members via social networking platforms or an online forum within the program website. This feature could potentially enhance user engagement as the integration of a chat room in web-based programs has been shown to improve social support scores [27]. An observational study also indicated that external motivators reduce dropout rates and increase success amongst participants [34]. Parents expressed that they would be more likely to participate in a program if it were endorsed by health professionals. Likewise, more than one third of the parents wanted a dietician to oversee the program. This reinforces that programs should be developed based on scientific evidence and approved by qualified researchers and professional bodies.

Limitations to the current study included that responses were from a small sample size, self-reported and may be subjected to bias. The data collected in this study were from a broad age range of children, more targeted information could be obtained in future studies once a specific child demographic was chosen to allow better tailoring of an intervention for age related activities. The sample in this study is not representative of the population as the majority of children in this study were self-reported to be from the healthy weight range BMI was calculated based on self-reported height and weight, the validity of online self-report of height and weight by parents of children has not been confirmed. Since participants were predominately mothers, the results may not reflect fathers’ responses, or families from differing ethnicities and socioeconomic backgrounds. Survey respondents comprised 4% indigenous, which is similar to the total Australian population [35]. The survey was conducted online which may have introduced bias in favour of the online program. However, this method was deemed to be the most effective at reaching a broader demographic within the limited timeframe available for this study. This method also increased the generalizability of the study, allowed participant anonymity, and reduced participant burden by allowing participants to complete the survey at times convenient to them.

## 5. Conclusions

Parents are interested in an online healthy lifestyle program for families and expressed a preference for the program website to be easy to access and user friendly, informal but with opportunities for tailored advice and goal setting. Future healthy lifestyle programs for families could include information on healthy recipes, healthy food portion sizes, healthy eating for children, and include both the parents and children as agents of change.

## References

[1] Waters E., de Silva-Sanigorski A., Burford B., Brown T., Campbell K., Gao Y., Armstrong R., Prosser L., Summerbell C. (2011). Interventions for preventing obesity in children. Cochrane Database Syst. Rev..

[2] Golan M. (2006). Parents as agents of change in childhood obesity-from research to practice. Int. J. Pediatr. Obes..

[3] Ho M., Garnett S.P., Baur L., Burrows T., Stewart L., Neve M., Collins C. (2013). Impact of dietary and exercise interventions on weight change and metabolic outcomes in obese children and adolescents: A systematic review and meta-analysis of randomised controlled trials. JAMA Pediatr..

[4] Knowlden A., Sharma M. (2012). Systematic review of family and home-based interventions targeting paediatric overweight and obesity. Obes. Rev..

[5] Burrows T., Warren J., Collins C. (2010). The impact of a child obesity intervention of child feeding practices. Int. J. Pediatr. Obes..

[6] Hawe P., Degeling D., Hall J. (2000). Evaluating Health Promotion.

[7] Davison K., Jurkowski J., Li K., Kranz S., Lawson H. (2013). A childhood obesity intervention developed by families for families: Results from a pilot study. Int. J. Behav. Nutr. Phys..

[8] The Telecommunucation Development Sector, I. ICT Facts and Figures—The world in 2015. http://www.itu.int/en/ITU-D/Statistics/Pages/facts/default.aspx.

[9] Burrows T., Khambalia A., Perry R., Carty D., Hendrie G., Allman Farinelli M., Garnett S.P., Mcnaughton S.A., Rangan A.M., Truby H. (2015). Great “app-eal” but not there yet: A review of iphone nutrition applications relevant to child weight management. Nutr. Diet..

[10] An J., Hayman L., Park Y., Dusaj T., Ayres C. (2009). Web-based weight management programs for children and adolescents: A systematic review of randomized controlled trials. Adv. Nurs. Sci..

[11] McCully S., Don B., Updegraff J. (2013). Using the internet to help with diet, weight and physical activity: Results from the health information national trends survey (hints). J. Med. Internet Res..

[12] Australian Communications and Media Authority Communications Report 2013–2014 Series. Report 1-Australians’ Digital Lives. http://www.acma.gov.au/~/media/Research%20and%20Analysis/Research/pdf/Australians%20digital%20livesFinal%20pdf.pdf.

[13] ABS (2009). Children's Participation in Cultural and Leisure Activities, Australia, (cat. No. 4901.0).

[14] Breton E., Fuemmeler B., Abroms L. (2011). Weight loss—There is an appfor that! But does it adhere to evidence infomred practices?. Transl. Behav. Med..

[15] Holley T., Collins C., Morgan P., Callister R., Hutchesson M. (2015). Weight expectations, motivations for weight change and perceived factors influencing weight management in young Australian women: A cross-sectional study. Public Health Nutr..

[16] ABS (2011). 2011 Census of Population and Housing.

[17] Cole T., Pan H. (2002). Lms Growth Computer Program, 2.12.

[18] Cole T., Bellizzi M., Flegal M., Dietz W. (2000). Establishing a standard definition for child overweight and obesity worldwide: International survey. BMJ.

[19] Hutchesson M.J., Rollo M.E., Krukowski R.B., Ells L., Harvey J., Morgan P.J., Callister R., Plotnikoff R., Collins C.E. (2015). eHealth interventions for the prevention and treatment of overweight and obesity in adults: A systematic review with meta-analysis. Obes. Rev..

[20] Hesketh K., Waters E., Green J., Salmon L., Williams J. (2005). Healthy eating, activity and obesity prevention: A qualitative study of parent and child perceptions in Australia. Health Promot. Int..

[21] Borra S.T., Kelly L., Shirreffs M.B., Neville K., Geiger C.J. (2003). Developing health messages: Qualitative studies with children, parents, and teachers help identify communications opportunities for healthful lifestyles and the prevention of obesity. J. Am. Diet. Assoc..

[22] Hart K.H., Herriot A., Bishop J.A., Truby H. (2003). Promoting healthy diet and exercise patterns amongst primary school children: A qualitative investigation of parental perspectives. J. Hum. Nutr. Diet..

[23] McLean K., Wake M., McCallum Z. (2007). Overweight in medical paediatric inpatients: Detection and parent expectations. J. Paediatr. Child Health.

[24] Doolen J., Alpert P.T., Miller S.K. (2009). Parental disconnect between perceived and actual weight status of children: A metasynthesis of the current research. J. Am. Acad. Nurse Pract..

[25] Pursey K., Stanwell P., Collins C., Burrows T. (2014). How accurate is web-based self-reported height, weight and body mass index in young adults?. J. Med. Internet Res..

[26] Schranz N., Olds T., Cliff D., Davern M., Engelen L., Giles-Corti B., Gomersall S., Hardy L., Hesketh K., Hills A. (2014). Results from Australia’s 2014 report card on physical activity for children and youth. J. Phys. Act. Health.

[27] Wantland D.J., Portillo C.J., Holzemer W.L., Slaughter R., McGhee E.M. (2004). The effectiveness of web-based *vs.* non-web-based interventions: A meta-analysis of behavioral change outcomes. J. Med. Internet Res..

[28] Delamater A., Pulgaron E., Rarback S., Hernandez J., Carrilo A., Christiansen S., Severson H. (2013). Web based family intervention for overweight children: A pilot study. Child. Obes..

[29] Tucker P., Irwin J.D., He M., Bouck L.M., Pollett G. (2006). Preschoolers’ dietary behaviours: Parents’ perspectives. Can. J. Diet. Pract. Res..

[30] Morgan P.J., Lubans D.R., Callister R., Okely A.D., Fletcher R., Burrows T.L., Collins C.E. (2010). The “healthy dads healthy kids” randomised controlled trial: Efficacy of a healthy lifestyle program for overweight fathers and their children. Int. J. Obes..

[31] Collins C., Okely A., Morgan P.J., Jones R.A., Burrows T., Cliff D., Colyvas K., Warren J.M., Steele J., Baur L.A. (2011). Long-term outcomes of the hikcups multi-site randomized trial: Efficacy of a parent-centred dietary-modification program, child-centred physical activity program or both in overweight children. Pediatrics.

[32] Morgan P., Collins C., Plotnikoff R.C., Callister R., Burrows T., Fletcher R., Okely A., Young M., Miller A., Lloyd A. (2014). The “healthy dads, healthy kids” community randomized controlled trial: A community-based healthy lifestyle program for fathers and their children. Prev. Med..

[33] Jones R., Price N., Okely A., Lockyear L. (2009). Developing an online program to prevent obesity in preschool-aged children: What do parents recommend?. Nutr. Diet..

[34] Hutchesson M.J., Collins C.E., Morgan P.J., Callister R. (2013). An 8-week web-based weight loss challenge with celebrity endorsement and enhanced social support: Observational study. J. Med. Internet Res..

[35] ABS (2011). Estimates of Aboriginal and Torres Strait Islander Australians, June 2011.

